# The Association of Olfactory Dysfunction With Depression, Cognition, and Disease Severity in Parkinson's Disease

**DOI:** 10.3389/fneur.2021.779712

**Published:** 2021-11-22

**Authors:** Ting-Chun Fang, Ming-Hong Chang, Chun-Pai Yang, Yi-Huei Chen, Ching-Heng Lin

**Affiliations:** ^1^Department of Neurology, Taichung Veteran General Hospital, Taichung City, Taiwan; ^2^School of medicine, National Chung Hsing University, Taichung City, Taiwan; ^3^Department of Neurology, Kuang Tien General Hospital, Taichung City, Taiwan; ^4^Department of Medical Education and Research, Taichung Veteran General Hospital, Taichung City, Taiwan

**Keywords:** Parkinson's disease, University of Pennsylvania Smell Identification Test, non-motor symptoms, anosmia, depression, cognitive impairment

## Abstract

**Background:** Non-motor subtypes of Parkinson's disease (PD) include the limbic, cognitive, and brainstem phenotype, which may have different pathological pathways with olfaction. In this work, we aim to clarify the association between olfactory dysfunction, depression, cognition, and disease severity in PD.

**Methods:** A total of 105 PD subjects were included and divided into anosmia and non-anosmic groups, using the University of Pennsylvania Smell Identification Test (UPSIT). All patients were evaluated with the movement disorder society unified Parkinson's disease rating scale (MDS-UPDRS), the Beck depression inventory (BDI)-II, and the Montreal cognitive assessment (MoCA).

**Results:** The BDI-II and UPSIT scores had a trend of reverse correlation without statistical significance (β-coefficient −0.12, *p* = 0.232). However, the odds ratio (OR) in anosmia was 2.74 (95% CI 1.01–7.46) for depression and 2.58 (95% CI 1.06–6.29) for cognitive impairment. For the MDS-UPDRS total and Part 3 score, the anosmia had a β-coefficient of 12.26 (95% CI 5.69–18.82) and 8.07 (95% CI 3.46–12.67), respectively. Neither depression nor cognitive impairment is associated with motor symptoms.

**Conclusion:** More severe olfactory dysfunction in PD is associated with cognitive impairment and greater disease severity. Depression in PD may involve complex pathways, causing relatively weak association with olfactory dysfunction.

## Introduction

Parkinson's disease (PD) is a neurodegenerative disease that involves both motor symptoms and non-motor symptoms. Although the current diagnostic criteria are still based on the motor deficits, non-motor symptoms emerging in different stages of PD are established and affect the quality of life (1, 2). Olfactory dysfunction, which presents in several neurodegenerative disorders, also acts as a prodrome of PD, and is considered a supportive criteria in the International Parkinson and Movement Disorder Society (MDS) Clinical Diagnostic Criteria for Parkinson's Disease (2). Given that olfactory dysfunction is hypothetically related to neurotransmitter dysfunction (3, 4), other non-motor symptoms such as cognitive dysfunction or depression may similarly correlate with olfactory dysfunction in PD. A proposed concept classifies the non-motor symptoms into three types: The limbic phenotype, in which Lewy bodies spread through olfactory route, presents with prominent anosmia initially and leads to depression, fatigue, pain, and weight loss; The cognitive phenotype, in which Lewy bodies deposit in the neocortex, presents with mild cognitive impairment, dementia, and apathy. The brainstem phenotype, in which Lewy bodies spread through brainstem route, presents with sleep and autonomic symptoms and late onset hyposmia (1). The three phenotypes indicate different pathological progression, but all involve olfactory dysfunction to various degrees. Previous studies have revealed the possible relationship between olfactory dysfunction, motor disability, and non-motor symptoms in PD (5, 6). Cognitive impairment, especially in some cognitive domains such as executive and visuospatial function, might be associated with worse olfaction in PD (7, 8), but the correlation between global cognition rating scales and olfaction is contradictory (6–9). Although the correlations between olfactory dysfunction and some psychiatric manifestations are reported in previous studies, there is still controversy about the association between depression and olfactory dysfunction (5, 7, 10). In our previous work, we compared the factors associated with depression in early diagnosed PD patients with disease duration >5 years, and it suggests that BDI-II score is less relevant to olfactory dysfunction (11). Moreover, the data for the relationship between olfactory dysfunction and PD disease severity is also inconsistent (5, 6, 12, 13). In this work, we try to clarify the association of olfactory dysfunction with depression, cognition, and disease severity in our general PD group.

## Methods

### Participants

All study participants were enrolled at the outpatient clinic in Taichung Veterans General Hospital from 2017. The patients who fulfilled the International Parkinson and MDS Clinical Diagnostic Criteria for Parkinson's Disease (2), and completed the University of Pennsylvania Smell Identification Test (UPSIT) were included in this study (14). Those who were taking antidepressants (for depression, neuropathic pain, insomnia, etc.) were excluded, in avoidance of the plausible effect of antidepressants on olfaction (15). Those who did not fulfill the MDS Clinical Diagnostic Criteria for Parkinson's disease during the follow-up at the outpatient clinic, or who could not cooperate for all the clinical assessments were excluded. Hundred and five patients were included in the study (40 women, 65 men). The mean age of the participants was 63.6 ± 9.1 years. The present work was approved by Taichung Veterans General Hospital Institutional Review Board/ Ethics Committee (No. CE16171B). All methods were performed in accordance with the Declaration of Helsinki guidelines and hospital regulations. All participants provided written informed consent.

### Clinical Assessment

#### Olfactory Function

The validated Taiwanese version of the UPSIT was used. In each test, there were 40 odorants embedded in “scratch and sniff” labels, and the participants were required, after releasing each odorant using a pencil tip, to smell and identify the correct answer among four choices (14). Total score was 40 in this test. Previous studies reveal that the mean UPSIT score is around 17–20 in PD (16–18). The participants were divided into two groups using the cut-off of 19, which also fits the normative cut-off score for anosmia according to the UPSIT guidelines (19–22). The group having more severe olfactory dysfunction (i.e., UPSIT score <19) was termed as anosmic group.

#### Cognition

To evaluate global cognition, the Montreal Cognitive Assessment (MoCA) was adapted because of its high sensitivity and specificity for detecting cognitive impairment, and as it is one of the scales which is validated for assessing global cognitive abilities in PD (23, 24). A MoCA score below 26 was considered to represent impairment of global cognitive abilities.

#### Depression

The Beck Depression Inventory-II (BDI-II) was used for mood evaluation (25). To avoid the discomfort from motor symptoms which might affect the result of the test, the patients were at “on-status” conditions at the time of test. Participants with depression were defined as having a BDI-II score ≥14.

#### Disease Severity of PD

The Movement Disorder Society Unified Parkinson's Disease Rating Scale (MDS-UPDRS) was used to assess symptoms associated with PD (26). There were four parts in the MDS-UPDRS scale to evaluate disease severity of PD including the cognition, mood, psychosocial status, daily activity, motor symptoms, and the side effect of treatment. The total score (UPDRS T) and the Part 3 score (UPDRS 3) representing the severity of motor symptoms were used for analysis.

### Statistical Analysis

Clinical characteristics in the groups were compared using *t*-test for continuous variables, including age and the UPDRS score. Non-parametric statistics with Kruskal–Wallis test was used for the BDI-II, the MoCA, and duration of the disease. The binary variables were analyzed by chi-square test. The odds ratios for depression and cognition impairment were calculated by binary logistic regression with covariates with age, gender, anosmia, and disease duration. We used linear regression to assess the relationship of UPSIT score with BDI-II and MoCA scores, and used multiple linear regression to assess the β-coefficient for UPDRS 3 with covariates including age, gender, anosmia, depression, impaired cognition and disease duration, and for UPDRS T with age, gender, anosmia, and disease duration.

All tests were two-sided with statistical significance level of 0.05, and were reported with *p* = 95% confidence intervals (CIs), or median and interquartiles. Statistical analyses were performed using SPSS (IBM Corporation, Armonk, New York, USA).

## Results

### Patient Characteristics

There were 105 PD participants in the study, with a mean UPSIT score of 17.63 (±6.59), who were divided into groups as follows: 57 in the anosmic group (24 women, 33 men), and 48 in the non-anosmic group (16 women, 32 men). There were no differences in gender, the BDI-II score, and disease duration between the two groups. Patients with anosmia were older (65.6 vs. 61.2, *p* = 0.013) and had lower MoCA score (25.0 vs. 27.0, *p* < 0.001) than the non-anosmic patients. There was a higher UPDRS T score (55.2 vs. 40.9, *p* < 0.001), as well as a higher UPDRS 3 score (33.1 vs. 23.5, *p* < 0.001) in anosmic patients (Table 1). When comparing depression and cognitive impairment, no significant relationship was noted (Table 2).

**Table 1:** Clinical characteristics of PD patients grouping with UPSIT cut-off of 19.

**Variable**	**Non-anosmia** **(UPSIT ≥ 19)**	**Total anosmia** **(UPSIT <19)**	***P*** **-value**

	***N*** **= 48**	***N*** **= 57**	
Age (year)	61.2 ± 9.5	65.6 ± 8.3	0.013
Gender (male), %	32 (66.6)	33 (57.8)	0.356
BDI-II	7.5 [3.2–12.0]	10.0 [4.0–17.0]	0.105^§^
MoCA	27.0 [25.2–29.7]	25.0 [21.0–28.0]	<0.001^§^
**MDS-UPDRS score**			
Part 3, motor symptoms	23.5 ± 10.8	33.1 ± 11.3	<0.001
Total	40.9 ± 14.7	55.2 ± 19.1	<0.001
Disease duration	3.0 [1.0–6.7]	5.0 [2.0–6.0]	0.306^§^

*Data were presented as mean ± standard deviation, or median [interquartiles]. UPSIT, University of Pennsylvania Smell Identification Test; BDI, Beck Depression Inventory; MDS-UPDRS, Movement Disorder Society Unified Parkinson's Disease Rating Scale; MoCA, Montreal cognitive assessment*.

§ *Analyzed with Kruskal–Wallis test*.

**Table 2:** Relationship between depression and cognitive impairment.

**Variable**	**Non-depression** **(BDI-II <14)**	**Depression** **(BDI-II ≥ 14)**	**χ^2^**	***P*** **-value**
	*N* (%)	*N* (%)	0.23	0.629
Impaired global cognition (MoCA <26)	29 (37.7)	12 (42.9)		
No cognitive impairment (MoCA ≧ 26)	48 (62.3)	16 (57.1)		

*BDI, Beck Depression Inventory; MoCA, Montreal cognitive assessment*.

### Association of Olfactory Dysfunction With Depression

The BDI-II score did not have a significant linear relationship with UPSIT score, but had a trend of reverse correlation with a β-coefficient of −0.12 (*p* = 0.232). Regarding olfactory dysfunction and depression as categorical variables, the anosmic group showed increased odds for depression with an odds ratio (OR) of 2.74 (95% CI 1.01–7.46, *p* = 0.047) (Table 3). The age, gender, and duration of the disease had no significant association with depression.

**Table 3:** Logistic regression results for depression (BDI-II ≥ 14).

**Variable**	**OR**	**95% CI of OR**	***P*** **-value**
Age	0.99	0.94–1.04	0.757
Gender (male)	1.04	0.41–2.61	0.923
Anosmia (UPSIT <19)	2.74	1.01–7.46	0.047
Impaired global cognition (MoCA <26)	1	0.38–2.64	0.989
Disease duration	1.07	0.92–1.25	0.334

*OR, odds ratio; CI, confidence interval; BDI, Beck Depression Inventory; UPSIT, University of Pennsylvania Smell Identification Test; MoCA, Montreal cognitive assessment*.

### Association of Olfactory Dysfunction With Cognition

There was a positive linear relationship between the MoCA score and the UPSIT score (β-coefficient of 0.21, 95% CI 0.10–0.33, *p* < 0.001). Regarding olfactory dysfunction and cognitive impairment as categorical variables, the anosmic group showed increased odds for global cognitive impairment with an OR of 2.58 (95% CI 1.06–6.29, *p* = 0.036) (Table 4). Aging also had higher odds for cognitive impairment with an OR of 1.06 (95% CI 1.01–1.12, *p* = 0.016).

**Table 4:** Logistic regression results for impaired global cognition (MoCA <26).

**Variable**	**OR**	**95% CI of OR**	***P*** **-value**
Age	1.06	1.01–1.12	0.016
Gender (male)	0.81	0.34–1.93	0.641
Anosmia (UPSIT <19)	2.58	1.06–6.29	0.036
Depression (BDI-II ≥ 14)	1.04	0.39–2.74	0.925
Disease duration	0.95	0.82–1.11	0.579

*OR, odds ratio; CI, confidence interval; BDI, Beck Depression Inventory; UPSIT, University of Pennsylvania Smell Identification Test; MoCA, Montreal cognitive assessment*.

### Association of Olfactory Dysfunction With Disease Severity of PD

Table 5 shows an increasing UPDRS 3 score in the anomic group with a β-coefficient of 8.07 (95% CI 3.46–12.67, *p* < 0.001). Duration of the disease is linked to increasing UPDRS 3 scores with a β-coefficient of 0.88 (95% CI 0.14–1.62, *p* = 0.020). Depression, impaired global cognition, age, and gender had no significant association with the UPDRS 3 score. In Table 6, the anosmic group was linked to a higher UPDRS T score (β-coefficient of 12.26, 95% CI 5.69–18.82, *p* < 0.001). Duration of the disease, as opposed to age or gender, was related to an increasing UPDRS T score (β-coefficient of 2.14, 95% CI 1.04–3.24, *p* < 0.001).

**Table 5:** Multiple linear regression results for MDS-UPDRS Part 3 score.

**Variable**	***B***	**95% CI of** ***B***	***P*** **-value**
Age	0.16	−0.08–0.41	0.193
Gender (male)	1.63	−2.74–6.02	0.461
Anosmia (UPSIT <19)	8.07	3.46–12.67	<0.001
Depression (BDI-II ≥ 14)	1.71	−3.20–6.62	0.492
Impaired global cognition (MoCA <26)	0.93	−3.81–5.67	0.697
Disease duration	0.88	0.14–1.62	0.02

*B, unstandardized regression coefficient; CI, confidence interval; MDS-UPDRS, Movement Disorder Society Unified Parkinson's Disease Rating Scale; BDI, Beck Depression Inventory; UPSIT, University of Pennsylvania Smell Identification Test; MoCA, Montreal cognitive assessment*.

**Table 6:** Multiple linear regression results for MDS-UPDRS total score.

**Variable**	***B***	**95% CI of *B***	***P*** **-value**
Age	0.28	−0.06–0.64	0.265
Gender (male)	1.86	−4.65–8.37	0.572
Anosmia (UPSIT <19)	12.26	5.69–18.82	<0.001
Disease duration	2.14	1.04–3.24	<0.001

*B, unstandardized regression coefficient; CI, confidence interval; MDS-UPDRS, Movement Disorder Society Unified Parkinson's Disease Rating Scale; UPSIT, University of Pennsylvania Smell Identification Test*.

## Discussion

Our study demonstrates that anosmia (i.e., UPSIT score <19) in PD is associated with cognitive impairment and greater disease severity, and has weak association with depression. We did not find a strong relationship between depression and cognitive impairment. This meets the concept of three non-motor PD subtypes proposed in a previous study, in which the scheme of pathological spread illustrates the role of the olfactory system in limbic, cognitive, and brainstem phenotypes, with distinct pathways between these three subtypes (1).

In the light of depression, there is a trend of negative linear relationship between the BDI-II score and the UPSIT score without statistical significance in this study. However, taking the olfactory dysfunction and depression as nominal variables, the anosmia is associated with depression in PD patients. The PD pathways of olfactory route and gut–brain transmission, both of which interact with the limbic system and brainstem nuclei relating to depression, may result in the discrepant association between olfaction and depression. We previously reported that PD patients who got depression after being diagnosed with PD had higher incidence of dementia, implying that depression in PD may be associated with different subtypes or spreading routes (27). Depression is a common non-motor symptom occurring either independently or with anxiety in PD (1, 28). As per the proposed non-motor subtypes in PD, the limbic phenotype involved the olfactory system with prominent anosmia. It spreads through the limbic cortex and causes the symptoms such as depression, anxiety, pain, and weight loss (1), while weight loss in PD may be related to mood, cognition, and different stages of the disease (29, 30). Previous studies revealed an association between depression and loss of smell, while serotonergic function loss in the olfactory bulb also supports the concept (3–5, 9, 31–33). This supports how monoaminergic deficits, including serotonergic and noradrenergic systems, are common causes of depression and olfactory dysfunction, and that the mutual effect of mood circuit and hyposmia may contribute to PD anhedonia (34, 35). On the other hand, the microbiota-gut-brain axis may also be relevant to depression (36). In our previous study discussing about constipation, depression, and olfactory dysfunction in PD patient with disease duration >5 years, we found association between constipation and depression, but no difference on the depression scale in terms of olfactory identification (11). One crosssectional study also reported no significant association between olfaction and mood scales, except psychosis (7).

The association between cognitive impairment and anosmia in PD is compelling, as our results coincide with the previous studies (8, 9, 37–41). Some cognitive domains such as executive and visuospatial function, instead of global cognition, might be associated with olfaction (7). Olfactory dysfunction is a prodromal non-motor symptom occurring decades before the onset of motor symptoms (16, 42, 43). The Lewy body pathology in the olfactory bulb and the anterior olfactory nucleus takes place in Braak stage 1 and affects the transmission of acetylcholine or dopamine (44). With propagation, Lewy bodies are found in cholinergic neurons of the nucleus basalis of Meynert, undermining the integrity of the efferent central cholinergic pathway. Imaging studies with acetylcholinesterase PET demonstrate that limbic cholinergic denervation contributes more to olfactory dysfunction than to nigrostriatal dopaminergic denervation (38, 45). Progressive cholinergic denervation might explain the link between olfaction and cognition, as positive correlations between odor-identification scores and acetylcholinesterase activity within the hippocampus and neocortex are similarly found in PD (38).

In our study, patients with PD and with anosmia had more severe motor symptoms independent of depression and impaired cognition than the nonanosmic group. This corresponds to the findings of other studies that there is a correlation between UPDRS and olfaction (5, 6). The substantia nigra (SN) is connected to olfactory structures, including the tubercle and entorhinal cortex, suggesting a pathway for **α**-synuclein transmission between the two regions (46). The relay pathway is complex, with retrograde caudo-rostral progression from the brainstem nucleus, or from anterograde rostro-caudal progression through the olfactory systems; the connection between the SN and olfactory structures may explain relevant motor symptoms and anosmia in PD. The link between dopaminergic neurons degeneration and olfactory dysfunction is unclear. Previous work shows positive correlation between striatal dopamine transporter (DAT) binding and UPSIT score, although the relationship may be stronger in the early motor stage of PD (5). In contrast, another study reports that DAT binding at the hippocampus, amygdala, and striatum have no correlation with olfactory tests in 29 PD patients (47). Treatment with levodopa or dopamine agonists are also ineffective in olfactory dysfunction (48), which may indicate that olfactory dysfunction is consequent to deficiency of multiple neurotransmitters in addition to dopamine. Yet, **α**-synucleinopathy is not a simple linear progression from olfactory system, but interconnects with the vagal retrograde route.

There were some limitations in this study. First, due to the crosssectional design, we were unable to address causality with anosmia, depression, cognitive impairment, and motor symptoms. Further follow-up studies would needed to be taken to elucidate the disease progression. Second, since using the UPSIT for olfactory assessment depends on participant cognitive function, such as naming and long-term memory, there might be an overestimation of olfactory dysfunction in those with severe cognitive impairment (38, 49). Although we had excluded those who could not complete the UPSIT for patient selection, bias related to cognitive impairment could not be completely ruled out. The participant might select the correct answer by chance (out of four choices) for each odor. This possibly resulted in an underestimation of olfactory dysfunction. Moreover, we did not conduct an additional survey for the etiology of olfactory dysfunction, such as other neurodegenerative diseases, infectious rhinitis, or allergic rhinitis. In this study, we excluded the subjects who were taking antidepressants for depression, neuropathic pain, insomnia, etc. Excluding PD patients taking antidepressants for depression may cause inadequate interpretation and selection bias. However, our finding suggests that we need to pay more attention to possibly coexisting depressive symptoms in antidepressant-naïve PD patients who have anosmia.

In conclusion, more severe olfactory dysfunction in PD is associated with cognitive impairment, and greater disease severity. Depression in PD may involve complex pathways, causing relatively weak association with olfactory dysfunction. There is no significant relationship between depression and impaired global cognition. Neither depression nor cognitive impairment is related to motor symptoms of PD. The pathways between olfactory systems and regions involved in cognitive impairment, motor symptoms, and depression may be divergent in the PD patients.

## Data Availability Statement

The raw data supporting the conclusions of this article will be made available by the authors, without undue reservation.

## Ethics Statement

The studies involving human participants were reviewed and approved by Taichung Veterans General Hospital Institutional Review Board/ Ethics Committee (No. CE16171B). The patients/participants provided their written informed consent to participate in this study.

## Author Contributions

T-CF, M-HC, and C-PY conceptualized the project. Y-HC and C-HL performed the data acquisition and analysis. T-CF wrote the first draft of the manuscript. M-HC critically reviewed the manuscript. All authors contributed to writing and revising the manuscript.

## Conflict of Interest

The authors declare that the research was conducted in the absence of any commercial or financial relationships that could be construed as a potential conflict of interest.

## Publisher's Note

All claims expressed in this article are solely those of the authors and do not necessarily represent those of their affiliated organizations, or those of the publisher, the editors and the reviewers. Any product that may be evaluated in this article, or claim that may be made by its manufacturer, is not guaranteed or endorsed by the publisher.
